# Draft Genome Sequences and Genome Characterization of Three Toxigenic and Two Nontoxigenic Clostridioides difficile Clinical Isolates from Florida, USA

**DOI:** 10.1128/mra.00151-23

**Published:** 2023-04-17

**Authors:** Ishani Wickramage, Joshua Heuler, Zhong Peng, Sally Alrabaa, Xingmin Sun

**Affiliations:** a Department of Molecular Medicine, Morsani College of Medicine, University of South Florida, Tampa, Florida, USA; b Department of Internal Medicine, Morsani College of Medicine, University of South Florida, Tampa, Florida, USA; University of Rochester School of Medicine and Dentistry

## Abstract

Draft genome sequences of five Clostridioides difficile clinical isolates were obtained in Florida, USA. Three isolates, designated TGH29 (sequence type 1 [ST1]/clade 2), TGH79 (ST11/clade 5), and TGH91 (ST35/clade 1), contained toxin-encoding genes. The two nontoxigenic strains were classified as TGH114 (ST109/clade 4) and TGH132 (ST15/clade 1). Antimicrobial resistance determinants and plasmids were detected and putative prophages predicted in some isolates.

## ANNOUNCEMENT

Clostridioides difficile infection (CDI), the most common healthcare infection in the United States and a potentially life-threatening cause of antibiotic-associated diarrhea (1), is one of the top five urgent antibiotic resistance threats in the United States (2). While the production of exotoxins, primarily TcdA and TcdB, as well as the binary toxin encoded by *cdtA* and *cdtB*, are the primary determinants of C. difficile virulence (3), nontoxigenic strains are an important reservoir of antimicrobial resistance (AMR) determinants (4) and are being explored as a preventive strategy against CDI (5). As a rapidly evolving organism with increasing incidence, severity, and recurrence of infections (3), understanding the genomic characteristics of novel C. difficile isolates is important for epidemiological surveillance and to identify novel therapeutic targets.

We obtained deidentified and exempted fecal samples from clinically symptomatic patients diagnosed with CDI in Tampa General Hospital (FL, USA) in 2016 (6). C. difficile was isolated by culture on cycloserine-cefoxitin-fructose agar, and PCR-based testing was then conducted for the presence of C. difficile-specific toxin genes. Following anaerobic culturing overnight at 37°C in brain heart infusion broth, 15% glycerol stocks were prepared and stored at −80°C, and genomic DNA was extracted using the Qiagen genomic DNA extraction kit. Whole-genome sequences were obtained using paired-end libraries and the sequencing-by-synthesis (SBS) Illumina HiSeq 3000 platform. Following quality assurance using FastQC (7), the reads were *de novo* assembled into contigs using Qiagen CLC Genomics Workbench 11.0.1 (8) using the following parameters: word size = 64, minimum contig length = 1000, mismatch cost = 2, and no scaffolding. The contigs were ordered against a reference genome (C. difficile strain 630; GenBank accession number CP010905.2) using Mauve 20150226 (9). The draft assembly was annotated using the NCBI Prokaryotic Genome Annotation Pipeline (PGAP) (10). The Center for Genomic Epidemiology multilocus sequence typing (MLST) 2.0 tool (11) was used for MLST and MLST phylogeny-based clade determination. AMR determinants were detected using the Comprehensive Antibiotic Resistance Database (CARD) 3.2.5 (RGI 6.0.0) (12), considering only perfect hits with 100% identity and minimum length thresholds. Intact prophages were predicted using *Pha*ge *S*earch *T*ool *E*nhanced *R*elease (PHASTER) (13), and the plasmid database PLSDB 2.1.1 (14) was used to detect plasmids, which were verified by NCBI BLASTn (MegaBLAST) sequence alignment (15). Using BLASTn (15), the genomes were also aligned with the genes *tcdA*, *tcdB*, *cdtA*, and *cdtB* of the strain 630 reference genome (CP010905.2) to search for toxin-encoding genes. Unless otherwise noted, default parameters were used for all programs.

The genome sequencing and assembly details, sizes, GC content, and PGAP-predicted number of genes and coding sequences (CDSs) are listed in Table 1. Using MLST, the three toxigenic isolates TGH29, TGH79, and TGH91 were categorized as sequence type 1 (ST1)/clade 2, ST11/clade 5, and ST35/clade 1, respectively. All three isolate genomes contained the full-length toxin genes *tcdA* and *tcdB*, while only TGH29 and TGH79 carried *cdtA* and *cdtB* genes encoding the binary toxin (Table 1). The two nontoxigenic strains TGH114 and TGH132 were designated as ST109/clade 4 and ST15/clade 1, respectively. The AMR determinants and plasmids detected and intact prophages predicted in all genomes are detailed in Table 1.

**TABLE 1:** Features of the Clostridioides difficile isolate genomes

Feature	Data for strain:				
	TGH29	TGH79	TGH91	TGH114	TGH132
Reads (Mb)	1,319	1,253	1,307	1,399	1,467
Genome coverage (×)	433	457	432	426	226
No. of contigs	62	51	69	63	70
N50 (bp)	146,560	140,974	214,318	147,410	170,898
Genome size (bp)	4,138,557	3,919,306	4,152,533	4,107,086	4,195,016
%GC	28.7	28.3	29.1	28.6	28.3
No. of genes	3,741	3,591	3,851	3,782	3,960
No. of CDSs	3,710	3,561	3,803	3,755	3,921
MLST	ST1	ST11	ST35	ST109	ST15
Clade	2	5	1	4	1
Toxin genes	*tcdA*, *tcdB*, *cdtA*, *cdtB*	*tcdA*, *tcdB*, *cdtA*, *cdtB*	*tcdA*, *tcdB*	-	-
AMR determinant(s) (resistance mechanism)	-^*a*^	*ant(6)-Ia* (aminoglycoside inactivation)	-	AAC(6′)-Ie-APH(2″)-Ia bifunctional protein (aminoglycoside inactivation)	CDD2 (carbapenem inactivation), *cdeA* (fluoroquinolone efflux)
Prophage features					
No. of predicted intact prophages	1	1	1	0	2
Prophage length(s) (kb)	55.2	50.6	63.8	-	33.9, 33.5
No. of open reading frames	69	78	85	-	40, 53
Most closely related phage(s)	PHAGE_Clostr_phiMMP01_NC_028883	PHAGE_Clostr_phiCDHM19_NC_028996	PHAGE_Clostr_CDMH1_NC_024144	-	PHAGE_Clostr_phiCD211_NC_029048, PHAGE_Clostr_phiCD506_NC_028838
Plasmid(s) detected (GenBank accession no.)	-	C. difficile strain 12038 plasmid unnamed (NZ_CP033215.1)	-	-	C. difficile strain 7032985 plasmid pCD-ISS1 (NZ_MG266000.1), C. difficile strain DSM 101085 plasmid unnamed (NZ_CP021320.1)

a -, none.

### Data availability.

The whole-genome shotgun projects for the toxigenic strains TGH29, TGH79, and TGH91 have been deposited at DDBJ/ENA/GenBank under the accession numbers JAPKMB000000000, JAPKMA000000000, and JAPKLZ000000000, the BioSample numbers SAMN31078169, SAMN31078170, and SAMN31078171, and the SRA accession numbers SRR21845281, SRR21845280, and SRR21845279, respectively, as well as the BioProject accession number PRJNA885148. The whole-genome shotgun projects for the nontoxigenic strains TGH114 and TGH132 have been deposited at DDBJ/ENA/GenBank under the accession numbers JAPKMD000000000 and JAPKMC000000000, the BioSample accession numbers SAMN31078188 and SAMN31078189, and the SRA accession numbers SRR21845362 and SRR21845361, respectively, as well as the BioProject accession number PRJNA885150. The versions described in this article are the first versions.
